# Relevance of Lipoprotein Composition in Endothelial Dysfunction and the Development of Hypertension

**DOI:** 10.3390/ijms26031125

**Published:** 2025-01-28

**Authors:** Lisette Monsibaez Ramírez-Melo, Diego Estrada-Luna, María Esther Rubio-Ruiz, Araceli Castañeda-Ovando, Eduardo Fernández-Martínez, Angélica Saraí Jiménez-Osorio, Óscar Pérez-Méndez, Elizabeth Carreón-Torres

**Affiliations:** 1 Nutrition Academic Area Instituto de Ciencias de la Salud, Universidad Autónoma del Estado de Hidalgo, Circuito Ex Hacienda La Concepción S/N, Carretera Pachuca-Actopan, San Agustín Tlaxiaca 42160, Hidalgo, Mexico; lisetterm21@gmail.com; 2 Nursing Academic Area, Instituto de Ciencias de la Salud, Universidad Autónoma del Estado de Hidalgo, Circuito Ex Hacienda La Concepción S/N, Carretera Pachuca-Actopan, San Agustín Tlaxiaca 42160, Hidalgo, Mexico; destrada_luna@uaeh.edu.mx (D.E.-L.); angelica_jimenez@uaeh.edu.mx (A.S.J.-O.); 3 Department of Physiology, Instituto Nacional de Cardiología Ignacio Chávez, Juan Badiano 1, Tlalpan, Mexico City 14080, Mexico; esther.rubio@cardiologia.org.mx; 4 Chemistry Academic Area, Instituto de Ciencias Básicas e Ingeniería, Universidad Autónoma del Estado de Hidalgo, Pachuca 42039, Hidalgo, Mexico; ovandoa@uaeh.edu.mx; 5 Medicine Academic Area, Instituto de Ciencias de la Salud, Universidad Autónoma del Estado de Hidalgo, Pachuca 42039, Hidalgo, Mexico; efernan@uaeh.edu.mx; 6 Department of Molecular Biology, Instituto Nacional de Cardiología Ignacio Chávez, Juan Badiano 1, Mexico City 14080, Mexico; oscar.perez.m@tec.mx; 7 Tecnológico de Monterrey, Campus Ciudad de México, Mexico City 14380, Mexico

**Keywords:** lipoproteins, endothelial dysfunction, hypertension, atherosclerosis, bioactive compounds

## Abstract

Endothelial dysfunction and chronic inflammation are determining factors in the development and progression of chronic degenerative diseases, such as hypertension and atherosclerosis. Among the shared pathophysiological characteristics of these two diseases is a metabolic disorder of lipids and lipoproteins. Therefore, the contents and quality of the lipids and proteins of lipoproteins become the targets of therapeutic objective. One of the stages of lipoprotein formation occurs through the incorporation of dietary lipids by enterocytes into the chylomicrons. Consequently, the composition, structure, and especially the properties of lipoproteins could be modified through the intake of bioactive compounds. The objective of this review is to describe the roles of the different lipid and protein components of lipoproteins and their receptors in endothelial dysfunction and the development of hypertension. In addition, we review the use of some non-pharmacological treatments that could improve endothelial function and/or prevent endothelial damage. The reviewed information contributes to the understanding of lipoproteins as vehicles of regulatory factors involved in the modulation of inflammatory and hemostatic processes, the attenuation of oxidative stress, and the neutralization of toxins, rather than only cholesterol and phospholipid transporters. For this review, a bibliographic search was carried out in different online metabases.

## 1. Introduction

Atherosclerosis is the leading cause of cardiovascular diseases (CVDs) worldwide [1]. Significant risk factors for heart disease include an unhealthy diet, physical inactivity, tobacco use, and the harmful use of alcohol. These risk factors are commonly present in individuals with high blood pressure, raised blood glucose, increased blood lipids, overweight, and obesity [2,3].

Chronic and low-grade inflammatory processes contribute to the onset of hypertension, likely due to physicochemical changes in the vascular microenvironment and increased oxidative stress. These changes cause substantial damage to the endothelium and increase the production of lipoperoxides, which exacerbate a pro-inflammatory, pro-thrombotic, and vasoconstrictive state [4] and endothelial dysfunction (ED) [5]. In this regard, lipids and lipoproteins play important roles in ED and the development of coronary heart disease. However, cardiovascular disease processes are multifactorial, and the molecular mechanisms involved still need to be further explored.

In addition, there are many compounds with different biological activities, such as anti-inflammatory, cholesterol-lowering, antibacterial, and antioxidant activities, which may reduce the development or prevalence of various chronic degenerative diseases, including cardiovascular diseases [6,7]. Vegetables and fruits are great sources of these bioactive compounds, including polyphenols, alkaloids, sterols, isothiocyanates, flavonoids, tannins, and terpenoids [8,9], as well as polyunsaturated fatty acids (PUFAs) [10]. Therefore, the development and prevalence of hypertension and atherosclerosis could be reduced by the consumption of these bioactive compounds. Certain nutraceuticals improve the composition, structure, and especially the functions of different lipoproteins [11,12]. In the same vein, it has also been suggested that the balance among different lipoproteins is necessary to exert a pro- or anti-atherosclerotic effect on endothelial cells and is not only limited to the transport of lipids [13]. Under abnormal conditions, lipoproteins are able to transport hydrophobic and amphipathic compounds, such as bacterial toxins, lipopolysaccharides (LPS), serum amyloid protein A (SAA), and oxidized lipids, among others [14,15]. Therefore, alterations in lipoprotein metabolism occur because of chemical and biophysical changes that promote or inhibit their function and the redistribution of nutrients to cells/tissues that could be damaged or repaired. Therefore, identifying lipids or other components of lipoproteins is essential to prevent damage and improve their function [16,17].

This article focuses on the impact of diverse lipoproteins and their miscellaneous components as therapeutic targets for maintaining the integrity and functionality of the vascular endothelium. The article also delves into the effects of lipoprotein-mediated endothelial dysfunction on the consequent development of some cardiovascular diseases, such as hypertension and atherosclerosis.

## 2. Relationship Between Lipoprotein Components and Endothelial Function

### 2.1. Lipoproteins

Plasma lipoproteins are heterogeneous macromolecular complexes composed of a highly hydrophobic core of non-polar lipids, including cholesterol esters (CEs) and triglycerides (Tgs), and a surface composed of apolipoproteins (apos) and amphipathic lipids, such as non-esterified cholesterol (free cholesterol, FC) and phospholipids (Phps).

Lipoproteins can be classified according to their flotation density, hydrodynamic diameter, sedimentation coefficient, apos content, and electrophoretic mobility (Table 1). In this context, seven classes of lipoproteins have been identified. The principal classification of these lipoproteins is based on their density and isolation by ultracentrifugation: chylomicrons (Cm), chylomicron remnants, very low-density lipoprotein (VLDL), intermediate-density lipoprotein (IDL), low-density lipoprotein (LDL), high-density lipoprotein (HDL), and lipoprotein (a) (Lp (a)) [18,19,20].

#### Lipoproteins Function

**Figure 1:**
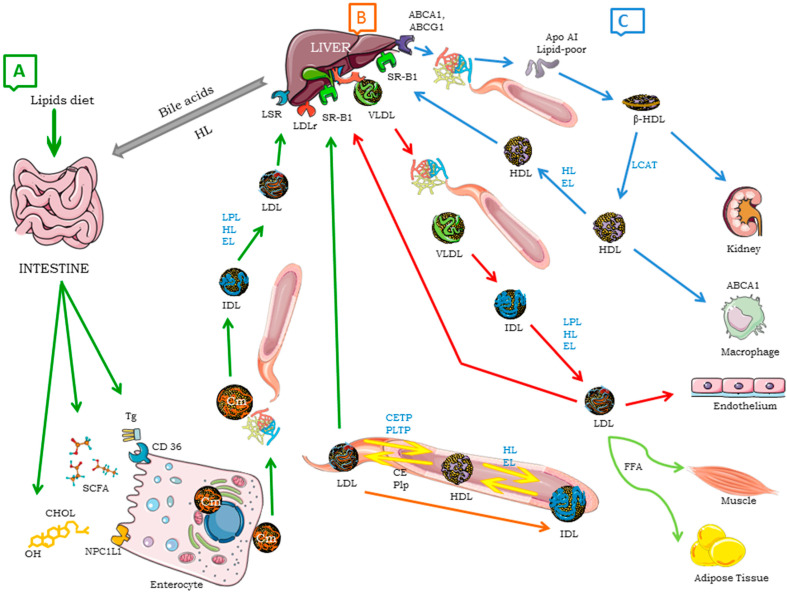
Lipoprotein metabolism. (**A**) The exogenous lipoprotein pathway (green arrows) begins in the gut lumina, where dietary lipids, such as Tgs and C, are incorporated into Cm. In enterocytes, cholesterol is captured through the NPC1L1 and then esterified by the action of the enzyme ACAT. The synthesized Cms are exocytosed by the basolateral membrane, allowing them to reach the lymphatic vessels. Subsequently, in the circulation, Tgs are hydrolyzed by the LPL to generate FFAs, and thus, they also acquire other components, such as apo E and apo C-II, from HDL. These FFAs are captured by various tissues, mainly by adipose tissue and striated muscle, where they are oxidized to produce energy or stored as Tgs. Cm remnants are then taken up by the liver by means of several receptors, such as LDLr, which binds to apo E, receptor LRP1, or the LSR. (**B**) The endogenous lipoprotein pathway (red arrows) initiates in the liver, where synthesized Tgs are packaged. Together with CEs, cholesterol, Phps, and apo B-100, they are assembled and secreted as VLDLs. VLDLs are metabolized by LPL and transformed into VLDLrs or IDLs, in addition to acquiring apo E and other apolipoproteins. Through the action of enzymes, such as HL, CETP, and PLTP, LDLs are converted into small and cholesterol-rich LDLs, which are taken up by LDLrs, LRP1, apo Er2, and VLDLrs through endocytosis in numerous tissues, including the liver, where apo B is fundamental for uptake. The LSR acquires affinity for apo E and apo B by associating with free fatty acids derived from triglycerides lipolysis. This contributes to the clearance of Qm remnants, triglyceride-rich lipoproteins, and LDLs [24]. (**C**) RCT or HDL metabolism (blue arrows) begins in the liver and intestine, with the synthesis of nascent HDLs, pre-β particles, and apo AI-poor lipid particles. In the circulation, the HDLs or pre-β particles are mediated by ABCA1, ABCG1, SR-B1, or passive diffusion. The lipidation process, as well as the conversion of FC to CEs, drives the formation of mature spherical α-HDL. Cholesterol esterification is mediated by LCAT, which catalyzes the transfer of a fatty acid from Phps to FC, resulting in the formation of CEs. Mature HDL undergoes constant remodeling through interactions with a variety of enzymes. Thus, cholesterol esters carried in the core of HDL particles can be transferred to apo B-containing particles in exchange for Tgs. This transfer is mediated by CETP and results in HDL enriched with Tgs, which can then be metabolized by lipases, such as HL, which hydrolyzes both triglycerides and phospholipids into HDL. Finally, HDL returns to the liver to restart the cycle.

Lipoproteins constitute the main lipid transport mechanism of dietary lipids absorbed by enterocytes. Lipoproteins also facilitate the transport of lipids from the liver to peripheral tissues, which is essential for obtaining energy and maintaining tissue function. Three transport mechanisms have been identified: (a) the trafficking of dietary lipids absorbed by enterocytes and directed to the liver [5,6,21]; (b) lipid transport from the liver to peripheral tissues and from peripheral tissues to the liver and intestine [7,8]; (c) HDL metabolism, known as reverse cholesterol transport (RCT) (Figure 1) [22,23,24]. This exchange of lipids (Tgs, cholesterol, CE, Phps, fatty acids) results in limitations on the size, composition, and function of different lipoproteins, especially LDL and HDL [25,26].

A pleiotropic function of lipoproteins is to transport hydrophobic and amphipathic compounds, such as bacterial toxins, LPS from Gram-negative bacteria, lipoteichoic acid from Gram-positive bacteria, SAA, and oxidized lipids, among others [14,15]. These components may affect the main function of lipoproteins, since they may be internalized by cells and deliver these hydrophobic molecules, inducing their damage or protection [27,28].

Therefore, it is important to highlight that the apolipoprotein and lipid composition of lipoproteins is not only remodeled under normal physiological conditions, but also in some pathologies. In this context, any condition that increases or decreases the absorption of free fatty acids will modify Cms and VLDLs. An abnormal influx of FA from adipose reserves into highly metabolic tissues leads to abnormal lipid accumulation, contributing to conditions such as intestinal dysbiosis, the postprandial state, inflammation, hypertension, metabolic syndrome, and diabetes [29,30,31]. Additionally, the FA composition of Tgs in Cms depends on the amount of fat ingested and the type of diet consumed [32]. It has been demonstrated that long-chain fatty acids promote LPS absorption from the gut and increase the plasma levels of bacterial endotoxins in humans [33].

Hence, alterations in lipoprotein metabolism cause chemical and biophysical changes that promote or inhibit their function and the redistribution of nutrients to cells/tissues, which could be damaged or repaired. Therefore, identifying the lipids and the other components of lipoproteins is essential for preventing damage and improving their function [16,17].

#### 2.2. Endothelium

The endothelium is a mechanical barrier that regulates the permeability of blood vessels and allows the passage of water and small molecules into the subendothelial space. In addition, it is a metabolically active organ responsible for several functions, such as the regulation of blood pressure. It is formed by negatively charged compounds that do not allow interactions between blood cells and the cell walls of blood vessels [34].

The endothelium may respond mechanically and molecularly to stimuli that regulate tone [35], hemostasis [34], and inflammation [36]. In normal physiological conditions, the endothelium does not proliferate, does not migrate, and minimally expresses adhesion markers. However, the endothelium is not inactive; it synthesizes a large number of molecules that allow it to fulfill all its functions [37,38]. Endothelial cells produce significant vasoactive molecules and maintain the vessel wall structure. Some of these molecules include the endothelium-derived relaxing factor, nitric oxide (NO), prostacyclins, the endothelium-derived hyperpolarizing factor, and the endothelium-derived contracting factor [34,39,40]. Other endothelial-derived vasoactive molecules include superoxide anion, endothelin-1, glycoproteins, proteoglycans, glycosaminoglycans, thromboxane, monocyte chemotactic protein-1 (MCP-1), granulocyte–monocyte colony stimulating factor (GM-CSF), and angiotensin-converting enzyme [41,42,43,44,45,46].

The dynamic and heterogeneous endothelium prevents the extravasation of different compounds, such as lipids, leukocytes, erythrocytes, platelets, and ions, among others. Additionally, the endothelial glycocalyx (eGlx) plays an important role in barrier function. The eGlx covers the luminal surface of endothelial cells and helps maintain the health of the vessel cells. Its alteration contributes to the pathogenesis of different diseases. There are numerous glycoproteins contained within the eGlx that provide a negative charge and prevent the contact of blood cells with the endothelium. Other molecules present as endothelial cell adhesion molecules, such as vascular cell adhesion molecule 1 (VCAM-1), intracellular adhesion molecule-1 (ICAM-1), E- and P-selectin, and components of coagulation and fibrinolytic cascade. The expression of glycoproteins is highly variable depending on endothelial cell activation [47]. The glycocalyx can also minimize damage to the endothelium by binding and activating superoxide dismutase, which in turn can neutralize oxidative stress caused by reactive oxygen species (ROS) [39]. Furthermore, adherens junctions, gap junctions, and tight junctions also contribute to the barrier function by maintaining the continuity of the endothelium and regulating endothelial permeability [48].

Therefore, maintaining the balance between vasodilation and vasoconstriction, anti-inflammatory and pro-inflammatory processes, and antioxidant and pro-oxidant activity, along with the generation of new blood vessels, is crucial to preserve the integrity of the endothelium and homeostasis of the cardiovascular system and avoid the progression of different pathologies, such as hypertension and atherosclerosis [36,41,49,50].

##### Endothelial Dysfunction Induced by Lipoproteins

Endothelial dysfunction is defined as the impaired function of endothelial cells caused by different stimuli that activate signaling pathways [49]. It is characterized by an increase in cytokine synthesis and the expression of endothelial markers, which allow for the recognition and extravasation or diapedesis of leukocytes, resulting in a pro-inflammatory state. ED begins with either (a) an imbalance in the production or bioavailability of endothelium-derived NO or (b) a change in the overproduction/impaired neutralization of free radicals or ROS, which increases oxidative stress. Furthermore, this imbalance promotes chemical and biophysical changes and causes substantial damage to the surfaces of membranes and lipoproteins [51].

It has been observed in sepsis, in CVD, and other pro-inflammatory states, that modifications of lipoprotein components contribute to the development of endothelial dysfunction [52]. For example, decreases occur in the HDL-cholesterol (HDL-C) and LDL-cholesterol (LDL-C) concentrations and in apolipoprotein A-I (apo A-I) and apo B [25,53]. Also, increases occur in lipoperoxide production, oxidized phospholipids (oxPLs), and lysophosphatidylcholine [54]. Additionally, during the postprandial state, circulating levels of triglyceride-rich lipoproteins promote changes in endothelial cells, causing vascular dilation, inflammation, and increased blood pressure [55,56]. The above suggests changes in the metabolism, synthesis, and catabolism of lipoproteins, as well as changes in their functionality. However, the mechanisms of action are not clear; it is uncertain whether lipoproteins modify the function of the endothelium, or if its dysfunction modifies lipoproteins.

In the process of endothelial function/dysfunction, there are not only modifications in the lipids of lipoproteins but also modifications in the rest of their components, including the apolipoproteins. These lipid-associated proteins, apos, participate in lipid internalization through cellular receptors, acting as cofactors of enzymes, as well as having anti-inflammatory, antioxidants, and anti-infective properties [25,57,58]. Several of these apos have been extensively characterized for their association with cardiovascular disorders and their presence in different lipoproteins [59,60]. It has been reported that in patients with type 1 diabetes mellitus, the risk of cardiovascular disorders can be predicted through modifications in the apo C-III content of their lipoproteins [61]. Several studies have shown a critical role of apolipoproteins in CVD, like atherosclerosis [62,63,64]. A deficiency of any of these proteins induces alterations in lipid metabolism, showing their fundamental role in the physiology of lipoproteins [65] (Table 2).

**Table 2:** Protein compositions of HDLs.

Apolipoproteins	Functions	Refs.
**Apo A-I**	Involved in the transport of cholesterol and other lipids.	[66,67,68]
	Involved the formation of new lipoproteins.	
	Antioxidant and anti-inflammatory properties.	
**Apo A-II**	The second most abundant protein in HDLs.	
**Apo A-IV**	Increased in the intestine during fat absorption.	
**Apo A-V**	Activator of lipolysis mediated by LPL; metabolism of lipoproteins rich in triglycerides.	
**Apo B**	Two related proteins, B-48 and B-100. Biomarker of cardiovascular risk and development of atherosclerosis. B-100 is large in size, has moderate hydrophobicity, and is unable to be transferred to other lipoproteins.	[69,70]
**Apo C**	Three different proteins; C-I, C-II, and C-III. Involved in the clearance of triglyceride-rich lipoproteins. C-III reduces the clearance of lipoproteins containing apo B and triglyceride-rich lipoproteins. Inhibits the binding of apo E and apo B to LDLr. Associated with HDL, inhibiting the apoptosis of endothelial cells and their capacity to inhibit monocyte adhesion to endothelial cells. A deficit of C-II increases Tgs, VLDL, and Cm while decreasing LDL, IDL, HDL, apo B, and apo A-I. Activates LPL.	[71,72,73]
**Apo D**	Also named lipocalin. Associated with lipid metabolism, inflammation, and antioxidative response. Involved in the transport of arachidonic acid. Modulates eicosanoid acid production. Provides neuroprotection.	[74,75,76]
**Apo E_2,3,4_**	Mediates the elimination of Cms and VLDLs. Helps to enrich the nuclei of HDLs with CEs. Reduced levels are related to hypercholesterolemia and atherosclerosis.	[77,78]
**Apo J**	Called “clusterin”. Protects cells against damage from oxidation, inflammation, and apoptosis. Associated with atherosclerosis, obesity, and diabetes.	[79,80]
**Apo M**	Involved in HDL metabolism and pre-β-HDL formation. Promotes the flow of cholesterol associated with HDLs. Anti-atherogenic, anti-inflammatory, and antioxidant effects. Primarily carries S1P.	[81,82,83,84]
**Apo (a)**	Binds to LDL-particles containing modified apo B-100.Pro-atherogenic, pro-inflammatory, and pro-thrombotic effects.Carrier of oxidized phospholipids.	[85,86,87,88,89]

HDL: high-density lipoprotein; Cm: chylomicron; VLDL: very low-density lipoprotein; IDL: intermediate-density lipoprotein; LDL: low-density lipoprotein; apo: apolipoprotein; LDLr: LDL receptor; LPL: lipoprotein lipase; S1P: sphingosine-1-phosphate.

Elevated levels of LDLs, VLDLs, and Cms and increased production of lipoperoxides stimulate vascular endothelial cells and smooth muscle cells to secrete MCP-1 and GM-CSF. This promotes monocyte chemotaxis, adhesion, and the differentiation of macrophages, which engulf and accumulate oxidized lipids (foaming cells). Additionally, it promotes the migration of smooth muscle cells to the subendothelial space and the development of fibrosis and necrosis [90]. Equally, advanced glycosylation end products, oxPLs, lysophosphatidylcholine, and pathogen-associated molecules are negative molecular stimuli for epithelial cells, prompting their dysfunction [91,92]. Likewise, high levels of plasma ceramides and sphingomyelins with specific long-chain fatty acids, which are present on the surface of HDLs or oxLDLs, and the binding of proteins that occurs in the acute phase of inflammatory processes, such as SAA, promote endothelial dysfunction [93,94,95].

The turnover of lipids from lipoproteins to the plasma membranes of tissues involves several types of lipoprotein receptors. There are two types: endocytic receptors, which bind to lipoproteins and mediate their internalization and subsequent lysosomal delivery, such as those of the LDLr family; and receptors, which bind to lipoproteins but transfer the lipid molecules directly to the plasma membrane without the cellular absorption of other component molecules of lipoproteins, such as type B scavenger receptors and lectin-like oxLDL scavenger receptor-1 (LOX-1). However, lipoprotein modifications can alter lipid turnover; for example, the modification of apo B through oxidation and glycation increases LDL uptake by CD36, scavenger receptors A (SRA)-I and SRA-II, and the lectin-like receptor family [86,96,97], which induces endothelial damage.

On the other hand, elevated levels of HDLs correlate with improved endothelial function due to their anti-atherogenic properties [98]. This protective capacity of HDLs has been attributed to their heterogeneity, highlighting HDL-associated proteins that have been identified [65,99]. However, HDLs can be easily modified and lose their cardioprotective properties through multiple mechanisms, similar to those of LDL oxidation [100]. Therefore, HDL cholesterol levels do not always predict function, and therefore, it is important to evaluate the quality and not just the quantity of HDL cholesterol.

**Figure 2:**
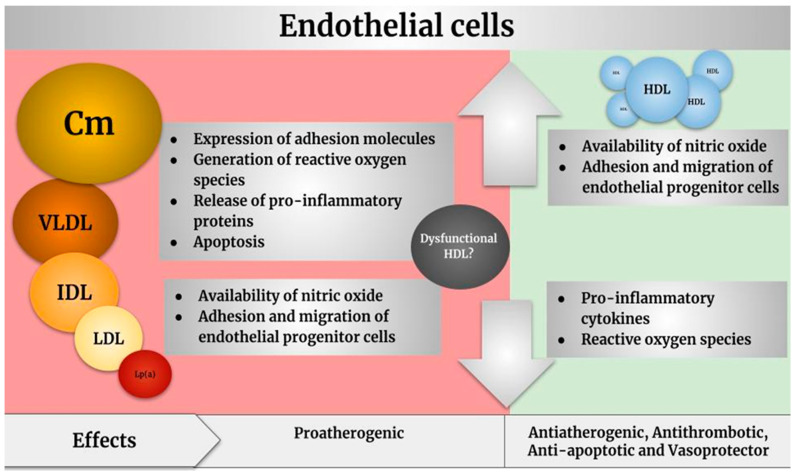
Effects of lipoproteins on endothelial cells. Cm: chylomicron; VLDL: very low-density lipoprotein: IDL: intermediate-density lipoprotein; LDL: low-density lipoprotein; Lp (a): lipoprotein (a); HDL: high-density lipoprotein.

The atheroprotective mechanisms associated with HDLs remain complex and little understood. Figure 2 summarizes the effects of lipoproteins on the endothelium described in this section.

## 3. Hypertension and Lipoproteins

Hypertension is a well-known major determinant for the development of atherosclerosis and has been considered a modifiable cardiovascular risk factor. However, control of hypertension and its complications has not been fully achieved yet, despite the currently available novel therapies. The etiology of hypertension is complex due to the interactions between environmental and physiological factors and genetic predisposition.

The endothelium plays an important role in regulating vascular tone and blood flow, thus supporting blood pressure (BP) control through the synthesis of a variety of vasoactive substances. Endothelial dysfunction occurs in association with several cardiovascular risk factors. It has been observed that about 60% of patients with hypertension develop atherosclerosis [101], accompanied by endothelial dysfunction, oxidative stress, vascular remodeling, fibrosis, and decreased immune regulatory T cells [5]. Hence, modern therapeutic strategies focus on preserving or restoring endothelial integrity.

In the relationship between the endothelium and BP, five mechanisms have been proposed: (1) increased aortic stiffness, (2) increased altered vascular tone due to an unbalanced production of vasoconstrictor/vasodilator factors, (3) oxidative and nitrosative stress, (4) increased inflammatory responses, and (5) increased endothelial–mesenchymal transition (EndoMT) [51].

NO is one of the most important molecules in the regulation of BP [86,102], and endothelial cells are responsible for producing this vasoactive substance. The deterioration of endothelial function in dyslipidemic patients may be caused by a reduction in the availability of NO, either due to a reduction in endothelial nitric oxide synthase (eNOS) activity mediated by oxLDL or to impaired NO metabolism [103]. In addition to the modulation of NO and ROS production, oxLDL induces the regulation of CAM expression on the endothelial surface and TNF-α secretion by inducing NF-kB [104]. The increase in oxLDL leads to an imbalance of eNOS, and inducible nitric oxide synthase (iNOS). This results in endothelial dysfunction due to endothelial cell apoptosis and reduced protective autophagy [105].

Additionally, LOX-1, an important receptor for the uptake of oxLDL, plays a crucial role in the generation of ROS in vascular smooth muscle cells (VSMCs). Recently, LOX-1 was reported to be associated with angiotensin II type 1 receptor (AT1R) and activate its ERK signal in endothelial cells. Therefore, they have been proposed as factors that contribute to the development of atherosclerosis, as well as to the damage found at the endothelial level, triggering inflammatory and pro-oxidant processes [106].

Moreover, it has been observed that HDL dysfunction is not only characterized by a loss or reduction of normal functionality but by increases in atypical functions. HDL in patients with chronic kidney disease inhibits NO production, rather than stimulating it, because of its ability to interact with the main receptor for oxidized LDL on LOX-1 receptor and toll-like receptors (TLR)-2 and -4 [107,108]. This regulation of LOX-1 occurs in response to an excessive accumulation of malondialdehyde (MDA), a highly reactive compound derived from lipid peroxidation. All these changes result in increased activation of the eNOS-inhibitor endothelial protein kinase C βII (PKCβII), ultimately reducing the capacity for endothelial cells to generate NO [107,109].

Another relevant element involved is paraoxonase-1 (PON1), a lactonase enzyme with peroxidase, arylesterase, and homocysteine–thiolactonase activities [106,110]. PON1 is bound to HDL in plasma, providing a certain antioxidant property in these lipoproteins. Experiments conducted in vitro with HDL isolated from healthy subjects showed that the inactivation of PON1 resulted in decreased phosphorylation of the serine 1177 residue of eNOS (an eNOS activation site), increased phosphorylation of threonine 495 residue (a deactivation site), and a subsequent decrease in the bioavailability of NO. This relationship is presumed to be mediated by the transformation of oxidative myeloperoxidase (MPO) [111].

Additionally, during the natural progression of atherosclerosis, it was reported that MPO led to post-translational modifications, resulting in lipoperoxidation and reactive lipid dicarbonyls, which diminish HDL-associated PON1 activity [112].

Hence, over the last decades, research has been conducted to increase gene expression and PON1 activity to decrease the size of atherosclerotic plaques, lower oxLDL concentrations, and regulate blood pressure and lipid profiles [113,114,115].

## 4. Pharmacological vs. No Pharmacological Therapy for Endothelial Dysfunction

Some pharmacological treatments have become available in the last 40 years to modulate lipid metabolism and triglycerides, LDL-C, and HDL-C levels. However, some of these treatments have not been successful in improving risk factors, such as hypertension and endothelial dysfunction. Furthermore, few clinical reports explain the changes related to the chemical composition of lipoproteins and pharmacological treatments, as well as their repercussions at the metabolic level [116,117,118]. In the last ten years, new therapies have been developed to improve the metabolism, structure, and especially the function of lipoproteins. Consequently, under certain conditions, the compositions of these lipoproteins can exert vascular protection or toxicity, highlighting the importance of the molecules contained within their structure.

In this context, pharmacological interventions to reach LDL-C goals or to treat patients with statin intolerance include monoclonal antibodies or small interfering RNA (such as inclisiran) to inhibit proprotein convertase subtilisin/kexin type 9 (PCSK9) and bempedoic acid (an adenosine triphosphate (ATP) citrate lyase inhibitor) [119,120,121]. These drugs exert the favorable effect of reducing pro-atherogenic particles, LDLs. This effect can probably reduce cardiovascular risk and improve endothelial function. However, these treatments require more clinical trials to support their effectiveness in improving risk factors, such as endothelial dysfunction and hypertension.

The development of hypertension, atherosclerosis, and other cardiometabolic diseases could be prevented by improving endothelial function. In this context, the synergistic treatment of hypertension and dyslipidemia could reduce the risk of developing a coronary event since, in hypertension, endothelial dysfunction accelerates the development of the deleterious consequences of dyslipidemia, and conversely, the remodeling of the vascular wall present in dyslipidemia complicates the course of hypertension. Of note, ED appears to be reversible in response to therapeutic interventions targeting risk factors [122]. The pharmacological treatment of specific risk factors and lifestyle modifications, such as quitting smoking, losing weight, changing the diet, and exercising, are effective in preventing atherosclerotic diseases [123]. However, some β-blockers have not demonstrated a better endothelial protective effect than other classes of antihypertensive drugs. The reversal of endothelial dysfunction is an important point to evaluate drugs consumed by individuals in the development of hypertension and atherosclerosis [124]. Also, some of the adverse effects shown in the use of hypolipidemic drugs, such as statins, bile acid sequestrants, and fibrates, are myopathy, gastrointestinal effects, and effects on liver function and toxicity [125,126,127]. Nevertheless, there is scientific and clinical evidence about the cardioprotective effects of natural products whose mechanism of action is regulating lipoprotein levels [128,129,130].

### Bioactive Compounds and Their Effect on Lipoproteins

Currently, studies have reported a correlation between nutrient intake and plasma lipid levels. These findings highlight the importance of choosing the correct diet to control lipid levels. Hence, changes in eating habits, in addition to weight loss, the prevention or reduction of lipid peroxidation, stress reduction, and physical exercise would be the most effective strategies to reduce and prevent cell and tissue damage and hypertension. One of the stages in lipoprotein formation occurs through the incorporation of dietary lipids into the chylomicrons of the intestinal mucosa. Enterocytes absorb dietary lipids, such as triglycerides, cholesteryl esters, and phospholipids, after enzymatic hydrolysis in the gut. In this context, the consumption of foods of plant origin, such as fruits, vegetables, whole grains, legumes, and nuts, have demonstrated benefits for overall health, including reducing the risk of chronic diseases, such as diabetes, obesity, and hypertension [131,132,133]. Additionally, it is estimated that 67% of modern medicines come from plant sources despite advances in the pharmaceutical industry [8,10]. Several recent studies on healthy foods and nutraceuticals that improve lipoprotein metabolism have shown benefits not only from fat-soluble vitamins but also from groups of compounds, such as phytosterols, terpenes, polyunsaturated fatty acids, and certain flavonoids (Table 3).

For example, the administration of antioxidants increases resistance to the oxidative modification of LDL and the pro-atherogenic transformation of these particles, such as lipophilic vitamins (vitamin A and vitamin E) [134], vegetable flavonoids [135], and other phytochemicals [136]. These compounds are transported into lipoproteins in the blood and function as antioxidants because they protect DNA, lipids, and other cellular components from oxidative stress [137]. Among the flavonoids researched, oxyresveratrol, found in mulberry branch extracts, has attracted attention. It has been shown to protect human umbilical vein endothelial cells (HUVECs) from oxLDLs by promoting the dissociation of Nrf-2 from the Keap-1/Nrf-2 complex, thereby activating the Nrf-2/HO-1 signaling pathway. This suggests its potential use in the prevention and treatment of atherosclerosis [138]. Clinical studies have shown that the intake of red fruits can lead to a reduction in LDL cholesterol levels and an increase in HDL cholesterol in patients with high lipid levels, type 2 diabetes, or metabolic syndrome [139].

A suggested mechanism involves alterations in the gut microbiota composition, mainly due to dietary lipids [140], which could modulate these changes at the gut level in species such as *Proteobacteria*, *Firmicutes*, *Bacteroidetes*, and *Actinobacteria*. In recent years, it was demonstrated that cardiovascular disorders (e.g., hypertension or atherosclerosis) are associated with dysbiosis [141]. Other widely recognized mechanisms include alterations in the chemical composition of HDLs. In general, it has been reported that diet constituents are able to modify the lipid and protein concentrations of HDLs, enhancing or preserving their anti-inflammatory, antioxidant, and lipid metabolism properties in animal models and humans [11,114,142]. This occurs due to either the participation of individual bioactive compounds or their synergistic action to mitigate the consequences or prevent the onset of concomitant diseases.

Until now, none of the tested antioxidants could form the basis of anti-atherosclerotic therapy [143].

**Table 3:** Bioactive compounds and their effects on diseases that involve endothelial dysfunction.

Bioactive Compound	Food Matrix	Dose/Concentration	Experimental Model	Effects and Mechanisms of Action	Refs.
Phytosterols	Plant stanols	2 g/day	Healthy volunteers	Reduced cholesterol absorption and LDL-C, apo B, and oxLDL levels.	[136]
	Plant sterols	1.6–2.5 g/day	Dyslipidemic subjects	Decreased triglyceride levels.	[144]
	Phytosterol esters (β-sitosterol) (Fitocor^®^)	Phytosterols (2.6 g/day) were prescribed and supplied in 650 mg gelatin capsules for 12 weeks	Patients >18 years, with LDL-c ≥130 mg/dL and <190 mg/dL and triglycerides <400 mg/dL.	Decreased total cholesterol, increased HDL-C, and reduced IDL.	[145]
	Plant sterols	400 mL of soy milk enriched with 1.6 g of phytosterols/4 weeks	Thirty-eight moderately hypercholesterolemic volunteers	Reduced endothelin-1 plasma concentration by 11%.Reduced total plasma cholesterol concentration, triglycerides, and apo B.	[146]
Terpenes	Reagent	Vitamin E: 10 mmol/L α-13′-OH and 5 mmol/L α-13′-COOH	THP-1 monocytes and human monocyte-derived macrophages	Decreased CD36 expression and oxLDL absorption.	[134]
	Iraqi *Cicer areitinum*	Terpenes (500 mg/kg)/56 days	Hyperlipidemic mice	Decreased levels of total cholesterol, triglycerides, LDL-C, and VLDL-C. Reduced ALT, AST, and ALP enzymatic activities also in total serum bilirubin levels. Increased levels of HDL-C.	[147]
	*Callistemon citrinus*	High-fat-sucrose diet + 1,8-cineole (0.88 mg/kg body weight), limonene (0.43 mg/kg body weight), α-terpineol (0.32 mg/kg body weight), and a mixture of the three terpenes/15 weeks	Obese rats	Reduced triglycerides levels, advanced oxidation protein products and hydroxyalkenals, a lipid peroxidation product. Restored levels of reduced glutathione.	[148]
Polyunsaturated fatty acids	Fish oil	Mice diet containing 19% fish oil alone, mice diet containing 19% fish oil + aspirin (via drinking water 30 mg/L)	COX-1 neo mice (a neomycin (neo)-resistant cassette inserted in COX-1 intron 10 to ensure hypomorphic expression of COX-1 gene)	Hypolipidemic and antihypertensive effects. Increased levels of omega-3 PUFAs, including EPA, DPA, and DHA. Anti-inflammatory effect. Decreased expression of adhesion molecules.	[149]
	Fish oil	Capsule omega-3 PUFA equivalent to 640 mg (520 mg DHA and 120 mg EPA)	Healthy subjects and subjects with a history of CVD	Decreased P-selectin expression and the percentage of platelet–monocyte aggregates.	[150]
	Purified omega-3	460 mg EPA and 380 mg DHA twice a day for 5 weeks	Hypertriglyceridemic patients	Decreased triglycerides and HDL-triglyceride plasma concentrations. Anti-inflammatory properties. Increase in endothelial function.	[12]
	Fish oil	12 g/day of EPA- or DHA-rich fish oil supplement (~4.8 g/d total EPA + DHA)	Healthy normolipidemic adults	Decreased plasma TG levels, as well as overall particle numbers of VLDLs and TG-rich lipoprotein subfractions. Increase in plasma levels of apo M.	[151]
	Fish oil	1 g fish oil capsules consumed for 8 weeks—370 mg of EPA and 230 mg of DHA, 3 times per day; total EPA + DHA = 1800 mg	30–74-year-old subjects with at least one cardiovascular risk factor (dyslipidemia, high blood pressure, diabetes, or smoking)	Increased large HDLs and reduced small HDLs and the non-esterified fatty acids in HDL (NEFAs-HDL) levels. Reduced CETP activity. Decreased apo CIII and increased apo CII and PON1.	[152]

IDL: intermediate-density lipoprotein, HDL-C: HDL cholesterol, LDL-C: LDL cholesterol, ALP: alkaline phosphatase, ALT: alanine transaminase, AST: aspartate transaminase, CETP: cholesteryl ester transfer protein, COX-1: cyclooxygenase-1, DPA: docosapentaenoic acid, EPA: eicosapentaenoic acid, NEFAs-HDL: non-esterified fatty acids in HDL, oxLDL: oxidized low-density lipoprotein, PON1: paraoxonase-1, PUFAs: polyunsaturated fatty acids, CVD: cardiovascular diseases.

## 5. Materials and Methods

A bibliographic search was performed in online metabases, including PUBMED, MEDLINE, and SCOPUS, using the terms “endothelial dysfunction”, “lipoprotein”, “hypertension”, “bioactive compounds”, “apolipoprotein”, “cardiovascular diseases”, “inflammation”. Only articles between the years 2000 and 2024 were considered. The original studies (randomized controlled and clinical trials), cohort studies, meta-analyses, review articles, and articles published by our groups were included. Screening of the manuscripts was performed by reading the titles, abstracts, or full texts based on the inclusion criteria. A total 152 articles were reviewed, which allowed us to highlight the importance of lipoprotein function on the endothelium and the prevention of hypertension. We also focused on the importance of consuming bioactive compounds, with an emphasis on endothelial dysfunction prevention. The search was conducted independently by three researchers, and each article was reviewed by each of the authors to discuss the evidence.

## 6. Conclusions

Lipoproteins are macromolecular complexes with heterogeneous lipids and proteins that determine the size, shape, and surface charge of these particles. The biological properties and effects of lipoproteins on health are associated with the presence, concentration, and quality of their lipid and protein components. The synthesis of lipoproteins is essential to maintain a balanced lipid metabolism. Any disruption in these processes may result in the development of various diseases. The relationship of lipoproteins with the development of different diseases, including atherosclerosis and hypertension, is mediated, at least in part, by the effect of lipoproteins on endothelial dysfunction and the subjacent inflammatory process. These lipoproteins might be imagined as vehicles of regulatory proteins mechanistically involved in the modulation of inflammatory and hemostatic processes and the attenuation of oxidative stress rather than as only cholesterol and phospholipid transports. Currently, research is focused on various components derived from functional foods that could modify lipoprotein composition while preserving their athero-protective properties.

Finally, the mechanisms of action of pharmacological and natural intervention, their role in endothelial protection, and the development of cardiovascular effects need to be further investigated. Also, the ability of lipoproteins to transport lipophilic bioactive compounds merits future studies.

## Figures and Tables

**Table 1:** Classification and compositions of lipoproteins.

Lipoproteins	Electrophoretic Mobility	Source of Synthesis	Flotation Density (g/mL)	Size (nm)	Main Chemical Composition	Main Apolipoproteins
**Cm**	Origin	Intestine	<0.940	>70	**Triglycerides 90–95%**, phospholipids 3–6%, cholesterol 1–3%, protein 1–2% *	**Apo B-48**, apo C-I, II, III, apo E, apo A-I, II, IV, V
**VLDL**	pre- β	Liver	1.006	30–70	**Triglycerides 45–65%**, phospholipids 15–20%, cholesterol 4–8%, protein 6–10% *	**Apo B-100**, apo C-I, II, III, apo E
**IDL**	β	Blood vessel	1.019	20–30	Triglycerides 20–30%, phospholipids 20–30%, **cholesterol 35–40%**, protein 15–20% *	**Apo B-100**, apo C-I, II, III, apo E
**LDL**	β	Blood vessel	1.063	19–23	Triglycerides 4–8%, phospholipids 18–24%, **cholesterol 50–60%**, protein 18–22% *	**Apo B-100**
**Lp (a)**	pre-β	Liver	1.085	21–26	Triglycerides 3–5%, phospholipids 19–21%, **cholesterol 40–45%**, protein 27–29% *	**Apo (a)**
**HDL** **(Subclasses** **2a, 2b, 3a, 3b, 3c)**	α	Liver, intestine	1.210	7.5–12.5	Triglycerides 2–7%, **phospholipids 26–32**%, cholesterol 20–25%, protein 45–55% *	**Apo A-I**, II, IV, apo C-I, II, III Apo D, apo E, apo M, apo J

Classification of lipoproteins based on their density and isolation by ultracentrifugation. Cm: chylomicrons; VLDL: very low-density lipoprotein; IDL: intermediate-density lipoprotein; LDL: low-density lipoprotein; Lp (a): lipoprotein (a); HDL: high-density lipoprotein; Apo: apolipoprotein. * dry mass percentage.

## Data Availability

Not applicable.

## Abbreviations

ABCA1/G1	ATP-binding cassette class A type 1/class G type 1
ACAT	Acyl-coenzyme A: cholesterol acyltransferase
Apos	Apolipoproteins
AT1R	Angiotensin II type 1 receptor
BP	Blood pressure
CE	Cholesterol ester
CETP	Cholesteryl ester transfer protein
Cm	Chylomicron
COX-1	Cyclooxygenase-1
CVD	Cardiovascular disease
ED	Endothelial dysfunction
eGlx	Endothelial glycocalyx
EndoMT	Endothelial–mesenchymal transition
eNOS	Endothelial nitric oxide synthase
ERK	Extracellular-signal-regulated kinase
FC	Free cholesterol
FFA	Free fatty acid
GM-CSF	Granulocyte–monocyte colony stimulating factor
HDL	High-density lipoprotein
HDL-C	HDL cholesterol
HL	Hepatic lipase
HUVECs	Human umbilical vein endothelial cells
ICAM-1	Intracellular adhesion molecule-1
IDL	Intermediate-density lipoprotein
iNOS	Inducible nitric oxide synthase
LCAT	Lecithin-cholesterol acyltransferase
LDL	Low-density lipoprotein
LDL-C	LDL cholesterol
LDLr	LDL receptor
LOX-1	Lectin-like ox-LDL scavenger receptor-1
Lp (a)	Lipoprotein (a)
LPL	Lipoprotein lipase
LPS	Lipopolysaccharide
LSR	Lipolysis-stimulated lipoprotein receptor
MCP-1	Monocyte chemotactic protein-1
MDA	Malondialdehyde
MPO	Oxidative myeloperoxidase
NF-kB	Nuclear factor kappa B
NO	Nitric oxide
NPC1L1	Niemann-Pick C1-like 1 protein
oxLDL	Oxidized low-density lipoprotein
oxPL	Oxidized phospholipid
Php	Phospholipid
PLTP	Phospholipid transfer protein
PON1	Paraoxonase-1
RCT	Reverse cholesterol transport
ROS	Reactive oxygen species
S1P	Sphingosine-1-phosphate
SAA	Serum amyloid protein A
SCFA	Short-chain fatty acid
SRA/B1	Scavenger receptor A/class B type 1
Tg	Triglyceride
TLR	Toll-like receptor
TNF-α	Tumor necrosis factor alpha
VLDL	Very low-density lipoprotein
VLDLr	Very low-density lipoprotein receptor
VSMCs	Vascular smooth muscle cells
